# One-dimensional well-defined peapod Co_x_Ni_y_@C nanocomposite hollow nanorod towards enhanced electromagnetic performance

**DOI:** 10.1007/s42114-026-01702-0

**Published:** 2026-03-02

**Authors:** Yan Liang, Jiahang Qiu, Mu Zhang, Xiaodong Li, Xudong Sun, Dianning He, Xiaochen Wang, Hanhui Lei, Terence Xiaoteng Liu, Bowen Liu

**Affiliations:** 1 Key Laboratory for Anisotropy and Texture of Materials (Ministry of Education), School of Materials Science and Engineering, Northeastern University, Shenyang, 110819 PR China; 2 The 966th Hospital of PLA Joint Logistics Support Force, Dandong, 118000 China; 3 School of Health Management, China Medical University, No.77 Puhe Road Shenyang North New Area, Shenyang, Liaoning 110122 China; 4 School of Engineering, Physics and Mathematics, Faculty of Science and Environment, Northumbria University, Newcastle Upon Tyne, NE1 8ST UK; 5 Department of Orthodontics, School and Hospital of Stomatology, China Medical University, Shenyang, 110001 China

**Keywords:** One dimensional, Pea pod-like structure, Oxidation resistance, High electromagnetic performance

## Abstract

Microwave absorbing materials (MAMs) not only achieve high electromagnetic absorption, but also have the characteristics of lightweight and oxidation resistance. This is a problem that needs to be solved urgently in some special fields. Therefore, we have done a series of experiments and prepared one-dimensional pea pod-like Co_x_Ni_y_@C absorbing material. Through the experimental results and previous work, we find that one-dimensional structure has great potential to achieve high absorption efficiency and lightweight, and can achieve good impedance matching at low filling rate, which is attributed to the enhancement of conductivity of one-dimensional structure and the interface polarization caused by core-shell structure, and the one-dimensional structure will further increase the heterogeneous interface area and enhance the interface polarization loss. The minimum reflection loss (RL_min_) and effective absorption width (EABD) of a series of pea pod-like Co_x_Ni_y_@C were − 48.4 dB and 4.8 GHz for Co_2_Ni_1_@C (12.3 GHz, 2.2 mm), and-43.2 dB and 5.8 GHz for Co_3_Ni_1_@C (9.5 GHz, 2.1 mm). Because of the addition of Ni, the formed NiO layer protects the alloy particles, which makes Co_x_Ni_y_@C have certain oxidation resistance. After being oxidized at 350 ℃ for 12 h in air, the RL_min_ and EABD of Co_x_Ni_y_@C remained at -41.2 dB and 5 GHz, respectively. This work shows the important potential of one-dimensional structure in lightweight of MAMs, and provides valuable insights for the subsequent research on oxidation resistance of MAMs.

**Supplementary Information:**

The online version contains supplementary material available at 10.1007/s42114-026-01702-0.

## Introduction

Due to the deep integration of microwave technology in the field of electronic signals and artificial intelligence devices, 5G communications, wearable electronic devices, global positioning systems, power semiconducting and other devices have flourished in the past decade [1–3]. The electromagnetic radiation generated threatens people’s health and information security [4].

Single component electromagnetic absorption materials such as ferrite and carbon materials: ferrite because of its low production cost, simple preparation method, has been widely studied and concerned [5–7]; Carbon materials have excellent electrical conductivity. Moreover, due to the emergence of special structures such as carbon nanotube [8, 9]and graphene [10], a wave of carbon materials in the field of MAMs has been set off [11]. However, due to the single loss mechanism, it often has defects such as limited application scenarios and overly thick coatings, making it difficult to meet the current requirements for absorbing materials (MAMS). Therefore, the design of composite MAMs with multi-loss mechanism, lighter weight and broadband absorption has become a new research trend [12].

The composite absorbing material is to compound the dielectric material with dielectric loss ability and the magnetic material with magnetic loss ability, so that the composite absorbing material has excellent impedance and outstanding absorbing capacity. At the same time, specific microstructure is also designed to further enhance the structural loss capacity of the material [13–16]. For example, the core-shell structure can be applied in many fields [17], and its multi-interface structure can enhance the interface polarization effect of the absorbing materia, and the multi-layer structure can increase the reflection times of incident waves to achieve the effect of enhancing the absorbing capacity of the material [18, 19]. Shi et al. prepared Fe@SiO_2_@C-Ni nanospheres with core-shell structure by solvothermal method [20]. The good composite of magnetic Fe and Ni particles with C and SiO_2_ dielectric shells makes the materials have excellent electromagnetic absorption performance, the RL_min_ can reach − 45.55 dB (2 mm). Similarly, Liu et al. prepared core-shell CoNi@SiO_2_@TiO_2_ nanoparticles by solvothermal method.,the RL_min_ is −46.7 dB(2.5 mm) [21]. To sum up, the composite MAMs with dielectric and magnetic materials have better performance than single component MAMs. However, at present, the application scenarios of MAMs often need to reduce the weight of absorbing coatings as much as possible, so the density of traditional spherical core-shell MAMs is a challenge in practical application. Therefore, researchers try to reduce the density of MAMs by various means, such as designing cavities in microstructure and using lightweight materials [22]. Another effective idea is to reduce the filling rate of MAMs in the substrate while maintaining the absorbing performance, so as to achieve the purpose of lightweight. Among many materials, anisotropic low-dimensional materials have great potential to achieve this goal.

A good three-dimensional conductive network can be formed by overlapping one-dimensional tubular structures. EHP (Electron-hopping), AICT (Aggregation-Induced-Charge-Transport) model and conductive network equation proposed by Cao et al. based on carbon nanotubes have been widely recognized, and can be used to describe the charge transport behavior in various low-dimensional composites at present [23]. Therefore, one-dimensional nanotubular materials can effectively improve the conductivity of materials. In order to balance impedance matching, one-dimensional MAMs will use lower filling rate and thinner coating thickness than three-dimensional MAMs in practical application [24–28]. In addition, large specific surface area is an important characteristic of one-dimensional materials, and when it is compounded with other materials, many heterogeneous interfaces will be produced, and the charges on these interfaces will produce significant interface polarization to obtain better electromagnetic loss ability [29]. Zhang et al. prepared the magnetic carbon nanotube Co-N-CNT structure, and analyzed the electron hologram spectrum of the heterogeneous interface [30], and found that a large number of positive and negative carriers were distributed around the heterogeneous interface, which proved the generation of interface polarization. In addition, there are many examples to study the effect of interfacial polarization, which provide effective evidence for the enhancement of microwave absorption by interfacial polarization relaxation [31–34].

With the development of high-speed aircraft and marine technology, MAMs need to be applied in more harsh environments, which puts forward higher requirements for the stability of MAMs [35]. At present, researchers have done a lot of research on the corrosion resistance of MAMs [36–40], but the exploration on the oxidation resistance of MAMs is relatively few. At present, the main ways to improve the oxidation resistance of MAMs are to use SiO_2_ for surface modification, or to use ceramic materials with excellent high temperature performance [41–43]. Another idea is to use an oxide layer to protect the magnetic metal inside. Zhang et al. prepared FeSiCr@Fe_3_O_4_ core-shell structure absorbing material by coating a layer of Fe_3_O_4_ on the surface of iron-based alloy powder in situ [44], which improved the stability of absorbing performance of the material. Similarly, alloys containing Ni can form an oxide layer on the surface, thus protecting the alloy particles inside and improving the oxidation resistance of the material.

In this work, we prepared pea pod-like one-dimensional Co_x_Ni_y_@C MAMs with self-adjusting atomic ratio for the first time, which provided a simple preparation method for individualized preparation of one-dimensional MAMs with different components. First, Cobalt-nickel double oxides (Co_x_Ni_y_-Dos) with different Co and Ni ratios, was prepared by microemulsion template method. Pea pod-like Co_x_Ni_y_@C nanotubes with different Co and Ni ratios were prepared by in-situ polymerization with a layer of phenolic resin coated on the oxide surface and then by carbothermal reduction in argon atmosphere. And the influence of different ratio of Co and Ni on electromagnetic properties of MAMs was investigated. At a low filling rate of 20%, due to the conductive network structure, the material can achieve good impedance and wave absorption performance. In a series of samples, the RL_min_ of Co_2_Ni_1_@C is −48.4 dB and the EABD is 4.8 GHz (12.3 GHz, 2.1 mm). The RL_min_ of Co_3_Ni_1_@C is −43.2 dB and the EABD of Co_3_Ni_1_@C is 5.8 GHz (2.1 mm). Moreover, due to the advantage of self-adjusting components, the composite of use functions is realized. In this work, the addition of Ni improves the antioxidant capacity of the material. Pea pod-like Co_x_Ni_y_@C still has good electromagnetic absorption performance after oxidation at 350 ℃ for 12 h. After oxidation, the RL_min_ and EABD of Co_3_Ni_1_@C remained at −41.2 dB and 5 GHz, respectively. Therefore, Pea pod-like Co_x_Ni_y_@C one-dimensional absorbing material not only realizes lightweight, but also has the ability to work in harsh environment, which can meet the working requirements of MAMs in special fields at present, and has certain application value and research potential.

## Experimental

Chemicals: Chemicals: cetyltrimethyl ammonium bromide (CTAB), cyclohexane (C_6_H_12_), pentanol (C_5_H_12_O), cobalt acetate tetrahydrate (CoC_4_H_6_O_4_ · 4H_2_O), nickel acetate tetrahydrate (NiC_4_H_6_O_4_ · 4H_2_O), oxalic acid dihydrate (H_2_C_2_O_4_ · 2H_2_O), formaldehyde (37%), resorcinol (C_6_H_6_O_2_), ammonia (aq 26%). The deionized water used in the experiment was self-made in the laboratory.

### Synthesis of Co_x_Ni_y_-DOs nanotubes

One-dimensional tubular double oxides were prepared by microemulsion template method, dissolved 2 g of CTAB in a mixture of 70 ml of cyclohexane and 3 ml of n-pentanol, and stirred for 30 min. Then added 4 ml 0.1 mol·L^− 1^ oxalic acid and stir for 1 h until the mixed solution becomes transparent. x mmol of cobalt acetate and y mmol of nickel acetate were added to 40 ml of deionized water to obtain a transparent solution., added 3 ml mixed solution into the transparent solution, stir for 12 h, centrifuge and collect the product, and dry at 80 ℃ for 12 h. The pink precursor was heat treated in muffle furnace at 500 ℃ for 2 h to obtain one-dimensional tubular Co_x_N_y_-Dos. Where x is: 4.5, 4, 3.6, 3; y is 1.5, 2, 2.4, 3. The Co_x_Ni_y_-Dos with different Co and Ni ratios were denoted as Co_3_Ni_1_-Dos, Co_2_Ni_1_-Dos, Co_3_Ni_2_-Dos and Co_1_Ni_1_-Dos respectively.

### Synthesis of Co_x_Ni_y_@C nanotubes

The prepared one-dimensional tubular Co_x_Ni_y_-Dos (0.2 g) was dispersed in the mixed solution of 140 ml absolute ethanol, 60 ml deionized water and ammonia (aq 26% 0.8 ml) by ultrasound, and adjust the pH value to around 11. Then resorcinol (0.6 g) and 37% formaldehyde solution (0.84 ml) were dissolved in the mixture, stirred by magnetic force for 12 h, the product was collected and dried to obtain the intermediate product Co_x_Ni_y_-Dos@RF. The one-dimensional Co_x_Ni_y_-Dos@RF nanotubes were carbonized at 750 ℃ for 3 h in argon atmospher to obtain Co_x_Ni_y_@C with different Co and Ni ratios, which were recorded as Co_3_Ni_1_@C, Co_2_Ni_1_@C, Co_3_Ni_2_@C and Co_1_Ni_1_@C respectively.

### Characterization

X-ray diffraction of Cu-Kα radiation sources were used to characterize phases and crystal structures, the test range was 10°−90°. Scanning electron microscope (JEOL JSM-7001 F), transmission electron microscope and high resolution electron microscope (JEM-2100 F) were used to observe the rod-like structure and the coating structure. The elemental composition was characterized by energy dispersion spectroscopy (EDS). Laser confocal Raman spectrometer (HORIBA JY HR Evolution) with an excitation wavelength of 532 nm. X-ray photoelectron spectroscopy (XPS, AXIS-SUPRA) was used to detect the surface chemical valence states.

The nanocomposite was mixed with paraffin wax in a ratio of 1:4, heated until the paraffin wax melted, pressed in a mold and cooled to form a coaxial paraffin rings. The paraffin ring was placed into the coaxial air line of vector network analyzer (VNA, E5080B, Keysight), and the test range is 2–18 GHz.

## Result and discussion

**Fig. 1:**
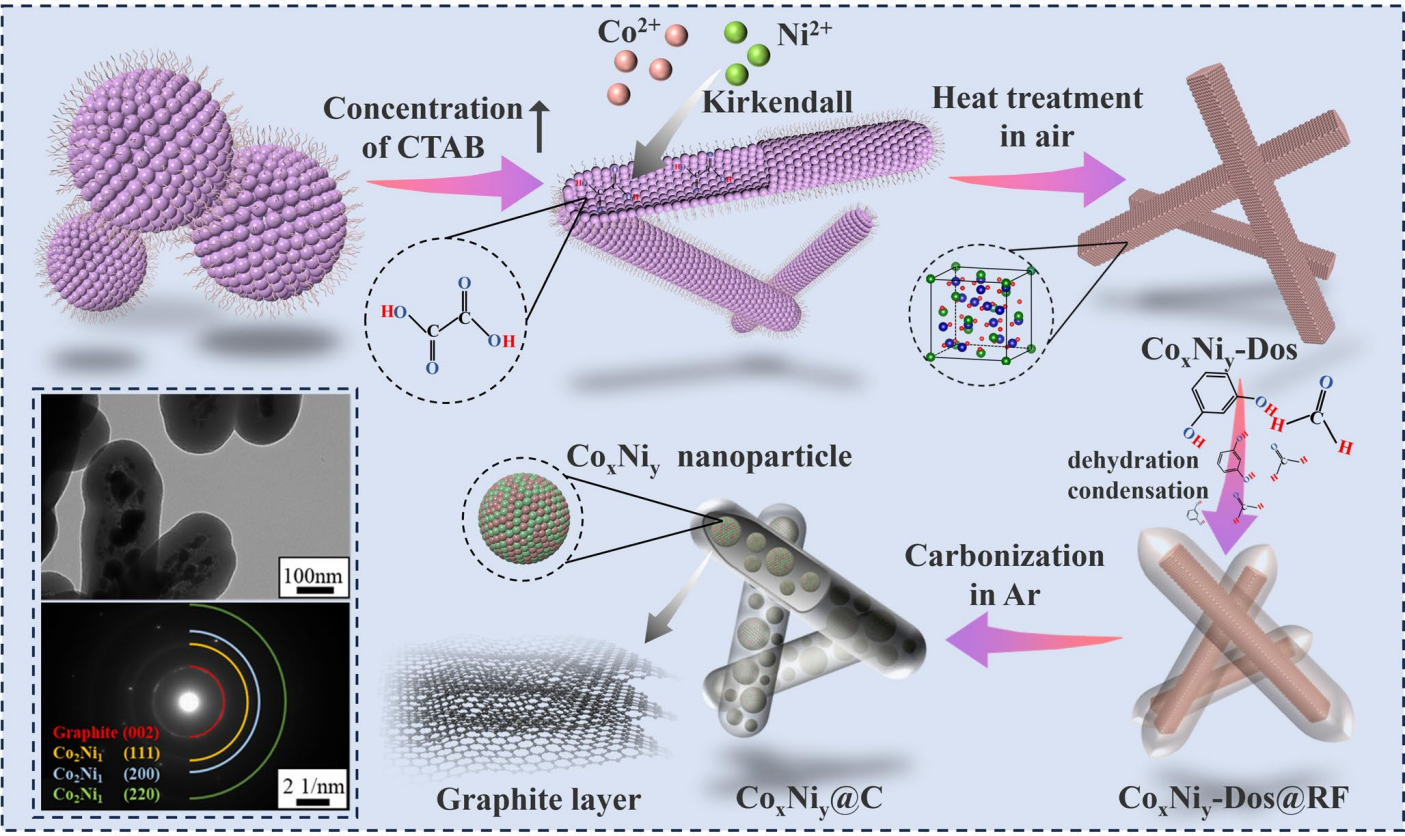
Schematic diagram of preparation and synthesis of pea pod-like Co_x_Ni_y_@C

Firstly, rod oxalic acid compounds were obtained by microemulsion template method, CTAB, as a cationic surfactant, formed micelles in the mixed solution due to the existence of lipophilic end and hydrophilic end. After reaching a certain concentration, under the action of van der Waals forces, the CTAB self-assembles into a rod-like structure. After adding different proportions of Co^2+^, Ni^2+^ and C_2_O_4_^2−^, the anion and cation precipitate in the rod-like capsule structure, and the rod-like oxalic acid compound is obtained. After that the oxalic acid compounds were calcined in air and oxalic acid compounds were decomposed and oxidized to form a rod-like one-dimensional oxide Co_x_N_y_-Dos. Based on rod-shaped oxide particles, the core-shell structure of Co_x_Ni_y_-Dos@RF was obtained by in-situ polymerization based on rod-liked oxide particles. After carbonization in argon atmosphere at high temperature, the oxides were reduced by C and CO produced by carbonization, and pea pod-like Co_x_Ni_y_@C was finally obtained. The principle of the preparation process is shown in Fig. 1.

**Fig. 2:**
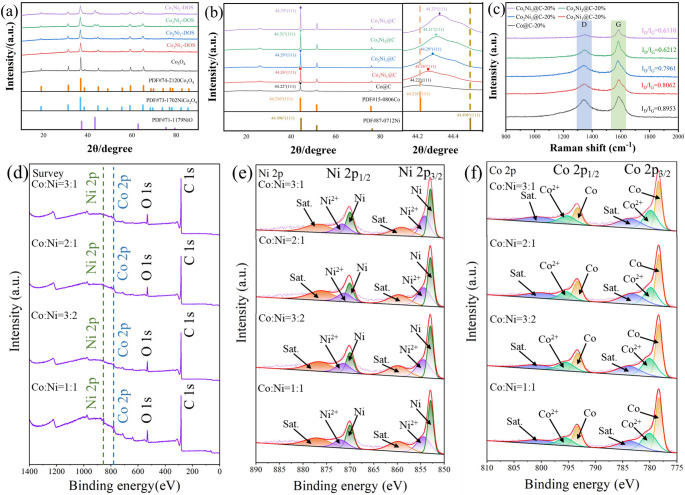
XRD diffraction patterns of (**a**) Co_x_Ni_y_-DOs nanotubes (**b**) pea pod-like Co_x_Ni_y_@C. Raman spectra (**c**) and XPS spectra of pea pod-like Co_x_Ni_y_@C: (**d**) survey scan, (**e**) Ni 2p, (**f**) Co 2p

XRD analysis of Co_x_Ni_y_-Dos and Co_x_Ni_y_@C was done. The XRD diffraction patterns of Co_x_Ni_y_-Dos with different Co and Ni atomic ratios are shown in Fig. 2a. It can be found that most of the oxides are double oxide phase with spinel structure (PDF # 73–1702) and contain a small amount of NiO (PDF#71–1179), it can be seen that the content of NiO increases gradually, which is related to the formation mechanism of oxides as cumulative nucleation. The XRD diffraction pattern of Co_x_Ni_y_@C is shown in Fig. 2b, in which the small peak at about 25° is the graphite peak produced by carbon graphitization. According to the standard cards (PDF#15–0806) and (PDF#87–0712), the strongest peaks of the (111) crystal plane of pure metal Co and pure metal Ni are located at 44.216° and 44.496°, respectively. The strongest peaks of one-dimensional Co@C of the prepared control sample correspond to the (111) crystal plane of Co. The strongest peaks of Co_x_Ni_y_@C with different Co and Ni ratios are respectively located in Co_3_Ni_1_@C (44.26°); Co_2_Ni_1_@C (44.29°); Co_3_Ni_2_@C (44.31°); Co_1_Ni_1_@C (44.33°). It can be seen that the peaks of all Co_x_Ni_y_@C (111) crystal planes are between 44.216° of pure Co and 44.496° of pure Ni, as shown in Fig. 2b, indicating that the sample has formed alloy phase. Moreover, with the increase of Ni content, the peak position of the (111) crystal plane of the sample gradually shifts towards the angle corresponding to the (111) crystal plane of pure Ni. This is because the atomic radius of Ni is smaller than that of Co, according to Bragg’s Law (2dsinθ = nλ) [45], where d represents the crystal plane spacing, θ is incident angle, and λ represents the wavelength of the ray. with the increase of Ni content, the interplanar spacing of (111) crystal plane gradually decreases, which leads to the diffraction peak of (111) crystal plane gradually shifting to a large angle.

In order to investigate the influence of the ratio of Co and Ni on carbon graphitization, Raman spectra of Co_x_Ni_y_@C were measured. When measuring the graphitization degree of carbon, we mainly focus on the vibration peaks at two positions (1350 cm^− 1^ and 1590 cm^− 1^). The former is called the D band; The latter is called the G-band, representing the sp^3^ hybridization of carbon atoms in defects and the sp^2^ hybridization in graphite hexagonal rings respectively. Therefore, the graphitization degree of carbon can be judged by the value of I_D_/I_G_. The lower the I_D_/I_G_ value, the higher the graphitization degree. The Raman spectrum of Co_x_Ni_y_@C is shown in Fig. 2c. The I_D_/I_G_ values are as follows, 0.8953 of Co@C, 0.8062 of Co_3_Ni_1_@C, 0.7961 of Co_2_Ni_1_@C, 0.6212 of Co_3_Ni_2_@C and 0.6110 of Co_1_Ni_1_@C. From this, it can be concluded, the graphitization degree increases with the increase of Ni content.

In order to investigate the surface valence state of Co_x_Ni_y_@C, X-ray photoelectron spectroscopy (XPS) analysis was carried out on the samples, as shown in Fig. 2d-f, it can be seen from the survey spectra that samples with different Co and Ni ratios have similar chemical states, all of which have C 1s, O 1 s, Co 2p and Ni 2p. As shown in Fig. 2e and f, in the spectra of Co 2p and Ni 2p, we can see spin-orbit splitting peaks of Co and Ni, which are located at 784.1 eV, 800.8 eV, 860.2 eV and 877.4 eV, respectively. At the same time, the characteristic peaks at 778.8 eV, 793.7 eV, 853.3 eV and 870.5 eV belong to Co and Ni metals respectively, and the characteristic peaks at 780.6 eV, 796.0 eV, 854.8 eV and 872.4 eV belong to Co^2+^ and Ni^2+^ respectively, indicating that a few metal particles are oxidized. Combined with the area comparison of Co and Ni metal peaks with Co^2+^ and Ni^2+^ characteristic peaks and XRD diffraction patterns, it can be analyzed that oxidation only occurs on the surface of particles, and the oxide layer protects the internal metal particles from further oxidation.

**Fig. 3:**
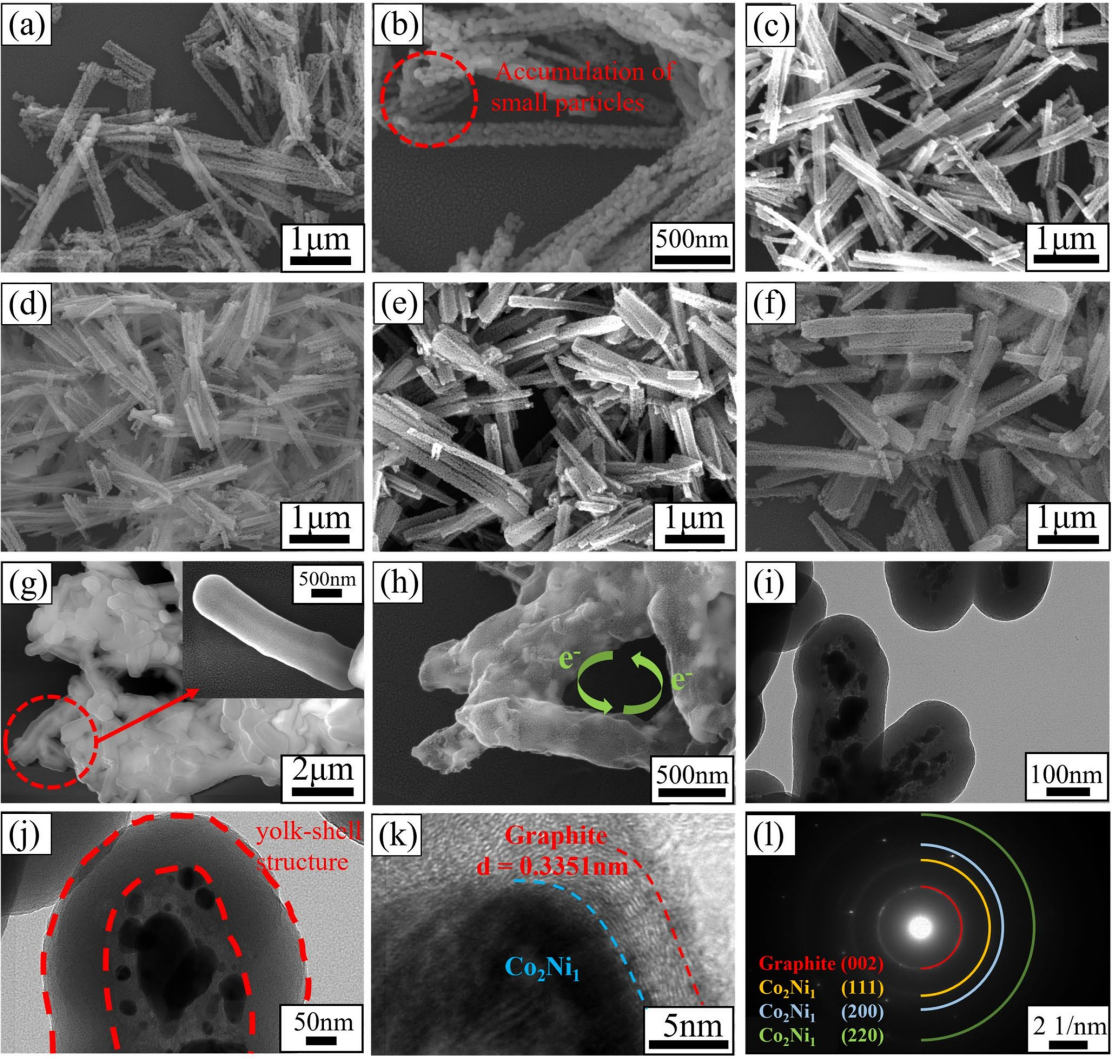
SEM images of Co_x_Ni_y_-DOs nanotubes: (**a**, **b**) Co_3_O_4_, (**c**) Co_3_Ni_1_-Dos, (**d**) Co_2_Ni_1_-Dos, (**e**) Co_3_Ni_2_-Dos, (**f**) Co_1_Ni_1_-Dos. (**g**) SEM images of Co_2_Ni_1_-Dos@RF. (**h**) SEM image of Co_2_Ni_1_@C. (**i**, **j**) TEM images, (**k**) HRTEM image, (**l**) SAED patterns of Co_2_Ni_1_@C

The morphology, element distribution and microstructure of Co_x_Ni_y_-Dos and Co_x_Ni_y_@C nanotubes were analyzed by SEM and TEM. It can be seen that the oxides have obvious one-dimensional tubular structure. Moreover, as can be seen from Fig. 3b, one-dimensional oxides are formed by accumulation of many small particles, which conforms to the accumulation nucleation mechanism mentioned above.

According to Fig. 3a-f, it is found that, the aspect ratio of CoNi-Dos decreases with the increase of Ni content, and gradually changes from slender tubular shape to shorter and thicker rod shape. Figure 3g shows the SEM morphology of phenolic resin coated one-dimensional oxide. Taking Co_2_Ni_1_@C as an example, Fig. 3h is the SEM image of Co_x_Ni_y_@C, in which the internal one-dimensional oxide is reduced to many metal particles, and the phenolic resin is carbonized, which can be seen more clearly through the TEM micrograph of Fig. 3i. It can be seen from Fig. 3j, pea pod-like Co_x_Ni_y_@C has an obvious core-shell structure Each one-dimensional tubular structure overlaps each other to form a three-dimensional network structure, which is beneficial to electron transmission and enhances the conductance loss of materials. Cao and coworkers [46] conducted a study on the electromagnetic attenuation behavior of multi-walled carbon nanotubes, and pointed out that when one-dimensional materials are sufficiently filled in the substrate, the hopping electrons between the nanotubes can enhance the microcurrent in the one-dimensional nanotube network, thereby forming a three-dimensional conductive network. This research provides effective support for the aforementioned statement. Through the HRTEM micrograph in Fig. 3k, it can be found that the carbon shell on the surface of metal particles is graphite layer and amorphous carbon, forming multiple heterogeneous interfaces of CoNi alloy/graphite/amorphous carbon. The interface polarization produced by these heterogeneous interfaces under the action of electromagnetic waves will significantly enhance the electromagnetic loss ability of materials. The SAED diffraction ring in Fig. 3l can also prove the existence of this interface, and each diffraction ring corresponds to the (002) crystal plane of graphite and the, (200), (220) and (111) crystal planes of CoNi alloy particles.

**Fig. 4:**
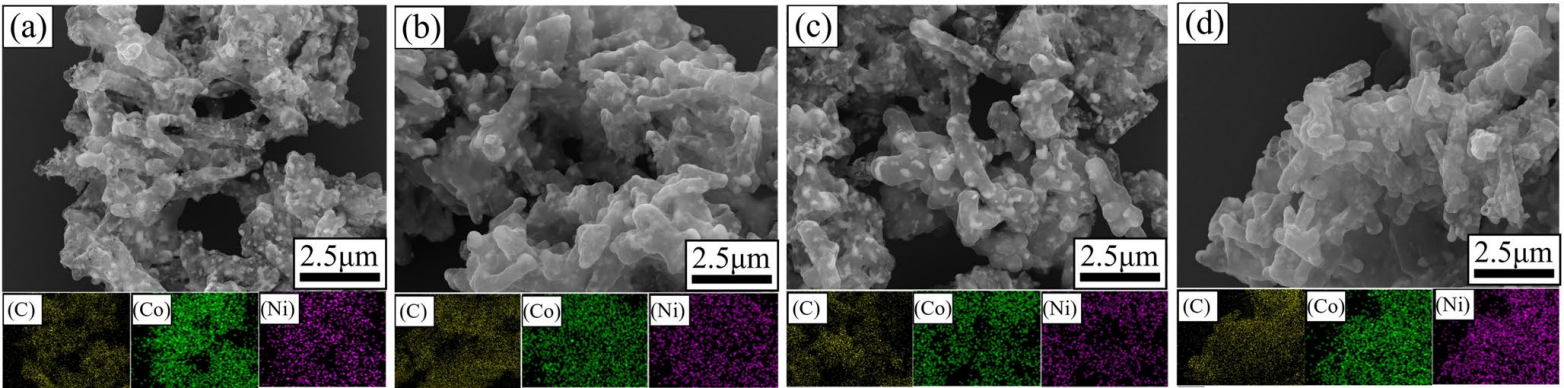
EDS elemental mapping of pea pod-like Co_x_Ni_y_@C with different atomic ratios of cobalt and nickel: (**a**) Co_3_Ni_1_@C, (**b**) Co_2_Ni_1_@C, (**c**) Co_3_Ni_2_@C, (**d**) Co_1_Ni_1_@C

It can be seen from EDS element distribution map that C, Co and Ni elements are evenly distributed. As shown in Fig. 4a-d, the atomic ratios of Co and Ni are 2.8, 2.0, 1.3 and 1, which basically conform to the designed atomic ratios.

**Fig. 5:**
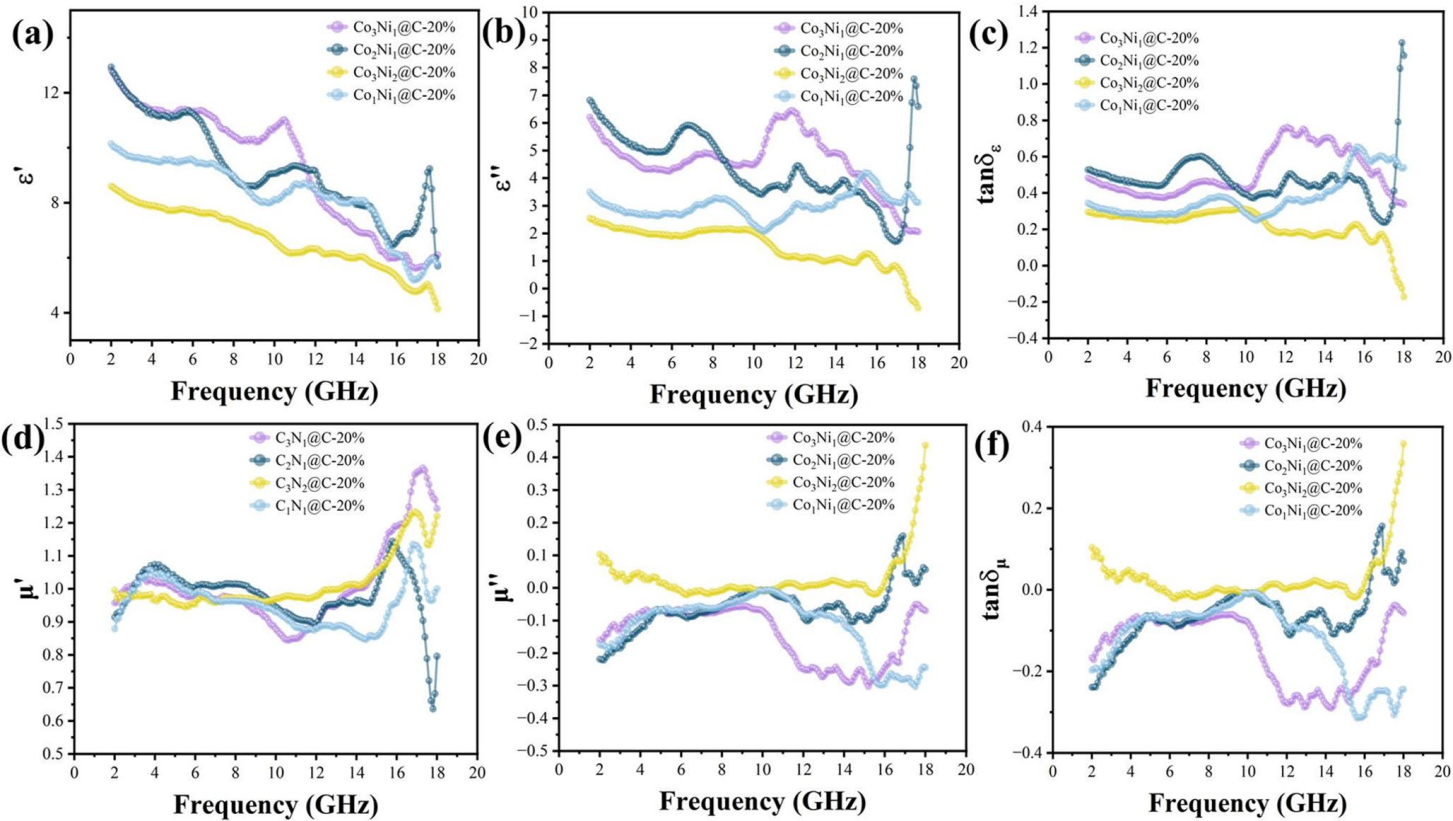
Electromagnetic parameter of pea pod-like Co_x_Ni_y_@C with different atomic ratios of cobalt and nickel: (**a**) ε’ curves, (**b**) ε’’ curves, (**c**) tanδ_ε_ curves, (**d**) µ’ curves, (**e**) µ’’ curves, (**f**) tanδ_µ_ curves

The electromagnetic performance of Co_x_Ni_y_@C with different Co and Ni ratios was tested by vector network analyzer. The result is shown in Fig. 5.

The permittivity ε = ε’-jε’’, and the permittivity decreases gradually with the increase of frequency, which accords with the dispersion phenomenon of electromagnetic waves. The ε’ decreases gradually with the increase of Ni content, but the ε’ of Co_3_Ni_2_@C is the lowest, as shown in Fig. 5a. Both Co and Ni particles play a catalytic role in the graphitization of carbon, and the graphitization degree of Co_x_Ni_y_@C increases gradually with the increase of Ni content. However, the aspect ratio of Co_3_Ni_2_-Dos is the smallest. When the surface of Co_3_Ni_2_-Dos is coated with phenolic resin in situ and heat treated, the aspect ratio of Co_3_Ni_2_@C should also be the smallest among different proportions of Co_x_Ni_y_@C. Therefore, when the one-dimensional tubular structure forms a conductive network, Co_3_Ni_2_@C is not as ideal as other proportions of Co_x_Ni_y_@C, which exceeds the positive effect of graphitization on permittivity, resulting in the smallest real part of permittivity. Figure 5b shows the ε’’ of Co_x_Ni_y_@C with different Co-Ni ratios, which represents the loss ability of MAMs to electromagnetic wave energy. Figure 5c shows the dielectric loss angle of Co_x_Ni_y_@C, and it can be seen that the ε’’ has the same change trend as the tanδε.

For the magnetic properties of Co_x_Ni_y_@C, it can be seen from Fig. 5d that in the range of 2–18 GHz, the µ’ is the general trend of slow decline. With the increase of frequency, at about 15 GHz, µ’ appears a sudden peak, showing a sudden increase phenomenon, which can be explained by exchange resonance and eddy current effect. Exchange resonance is caused by the exchange of magnetic energy between magnetic domains at higher frequencies. Exchange resonance is related to spin wave excitation, small size effect, and surface effect of ferromagnetic materials. When the size of nanoparticles decreases, their small size enhances the surface anisotropy, thereby making the exchange resonance phenomenon more significant [47, 48]. Among them, Co_3_Ni_2_@C has higher µ’’, which shows that it has higher magnetic loss ability. In Fig. 5e, we can find that µ’’ at the partial frequency of Co_x_Ni_y_@C has a negative value, according to Faraday’s law of electromagnetic induction, it can be explained that under the action of electromagnetic wave changing magnetic field, ferromagnetic materials produce eddy current effect, which induces a magnetic field opposite to the outside, showing diamagnetism, resulting in the negative value of µ’’. In Fig. 5f, the tanδ_µ_ and the µ’’ have the same change trend. According to Fig. 5c and f, Co_x_Ni_y_@C is a kind of absorbing material with dielectric loss as its main loss mechanism. Magnetic particles provide part of magnetic loss, and at the same time, their main function is to balance the impedance at lower frequencies.

The RL value of Co_x_Ni_y_@C can be calculated using transmission line theory, which can be expressed by electromagnetic parameters as [49]:

1 $$\:{Z}_{in}={Z}_{0}\sqrt{{\mu\:}_{r}/{\epsilon\:}_{r}}\mathrm{t}\mathrm{a}\mathrm{n}\mathrm{h}\left[j\right(2\pi\:fd/c\left)\sqrt{{\mu\:}_{r}/{\epsilon\:}_{r}}\right]$$

2 $$\:RL\left(dB\right)=20\mathrm{l}\mathrm{o}\mathrm{g}\left[\right({Z}_{in}-{Z}_{0})/({Z}_{in}+{Z}_{0}\left)\right]$$

where $\:{Z}_{in}$ and $\:{Z}_{0}$ are the input impedance and free space impedance of the absorbent respectively, f is the frequency, d represents the thickness of the absorber, and c velocity of light. The reflection loss value (RL) of absorbent is generally negative, which represents the loss degree of electromagnetic wave energy by MAMs, and-10 dB represents 90% absorption of electromagnetic wave energy. And the frequency range with RL less than − 10 dB is generally defined as the effective absorption width (EABD) of the absorbent. The lower the reflection coefficient R is, the less part of the electromagnetic wave is reflected. The R value of an ideal absorbent should be close to 0, and the expression of R value and impedance coefficient Z is as follows [50]:

3 $$\:R={(Z}_{in}-{Z}_{0})/{(Z}_{in}+{Z}_{0})$$

4 $$\:Z=\:{Z}_{in}/{Z}_{0}$$

**Fig. 6:**
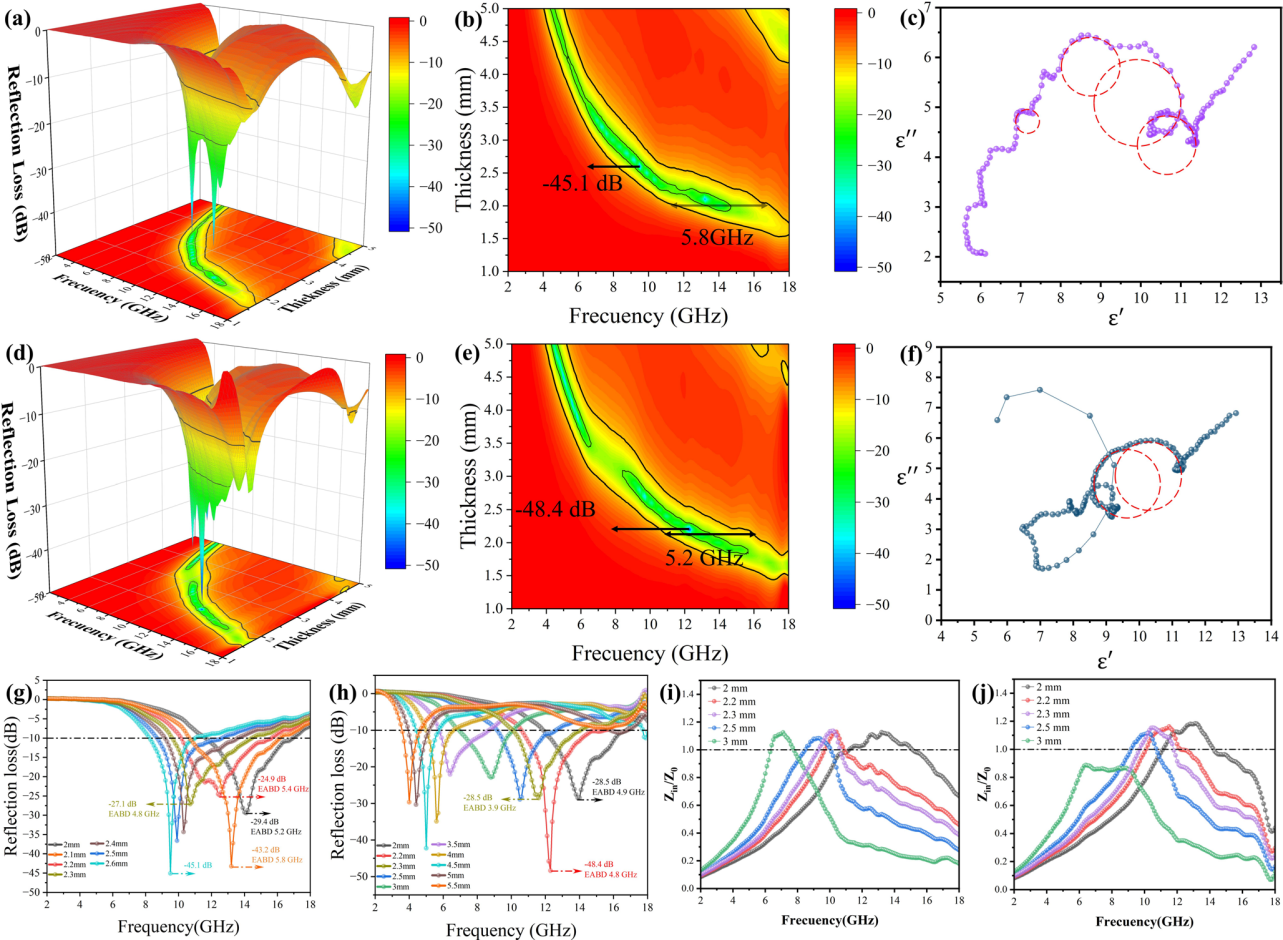
RL contour of pea pod-like Co_x_Ni_y_@C with different atomic ratios of cobalt and nickel: (**a**) Co_3_Ni_1_@C, (**d**) Co_2_Ni_1_@C. 3D RL maps of (**b**) Co_3_Ni_1_@C, (**e**) Co_2_Ni_1_@C. Cole-Cole plots of (**c**) Co_3_Ni_1_@C, (**f**) Co_2_Ni_1_@C. 2D RL plots of (**g**) Co_3_Ni_1_@C, (**h**) Co_2_Ni_1_@C. Impedance matching curves of (**i**) Co_3_Ni_1_@C, (**j**) Co_2_Ni_1_@C

**Fig. 7:**
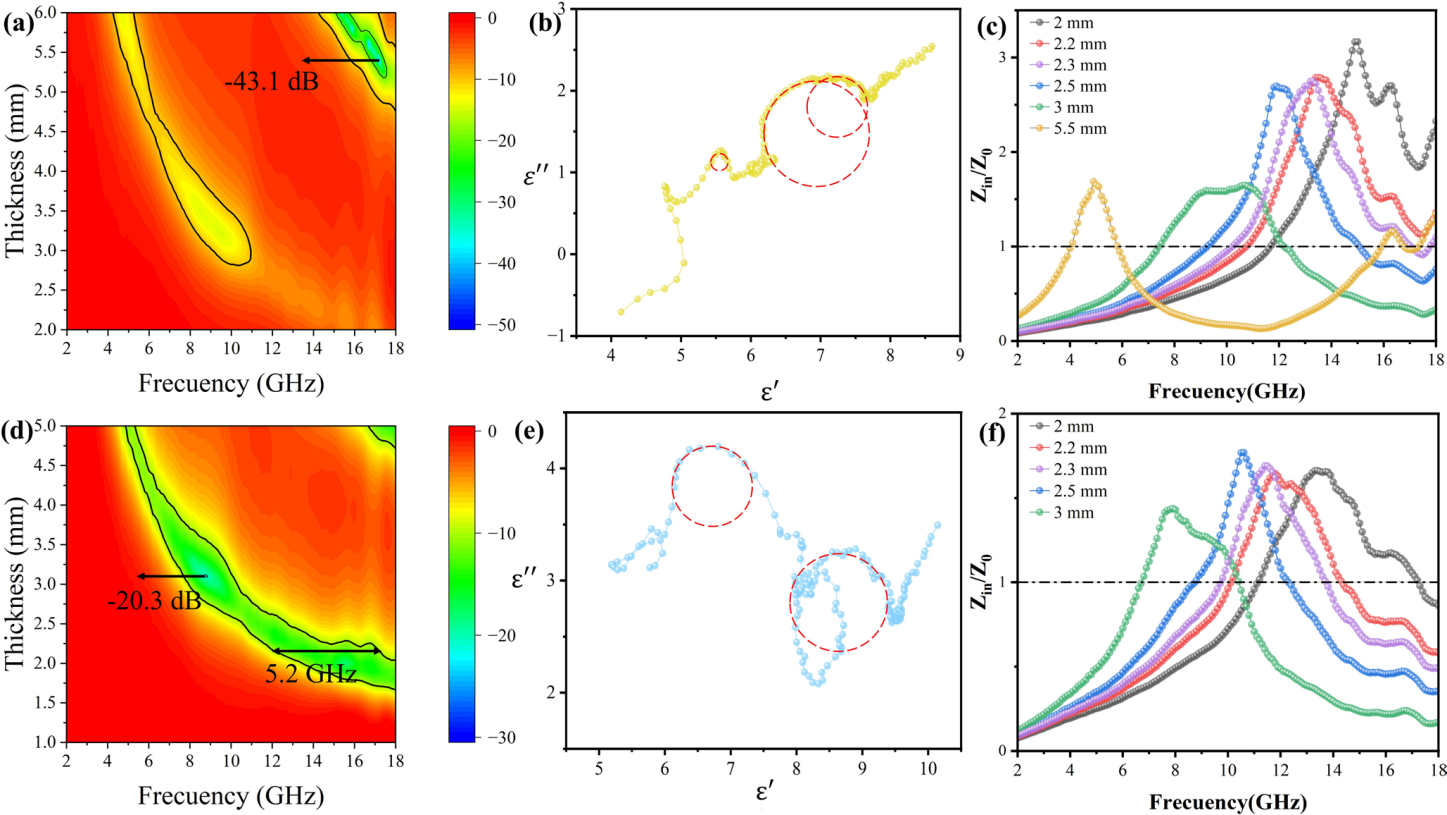
3D RL maps of (**a**) Co_3_Ni_2_@C, (**d**) Co_1_Ni_1_@C. Cole-Cole plots of (**b**) Co_3_Ni_2_@C, (**e**) Co_1_Ni_1_@C. Impedance matching curves of (**c**) Co_3_Ni_2_@C, (**f**) Co_1_Ni_1_@C

It can be seen that when *R* = 0, Z_in_=Z_0_ is required, and the impedance coefficient Z = 1. At this time, the absorbent has good impedance matching, which can achieve good microwave absorption at the frequency and thickness corresponding to Eq. (1). The diagram of impedance matching is shown in Figs. 6 and 7.

The ε’ and ε’’ can be derived from Debye’s free electron theory to be [51]:

5 $$\:{\epsilon\:}^{{\prime\:}}={\epsilon\:}_{\infty\:}+\frac{{\epsilon\:}_{s}-{\epsilon\:}_{\infty\:}}{1+{\omega\:}^{2}{r}^{2}}$$

6 $$\:{\epsilon\:}^{{\prime\:}{\prime\:}}={\epsilon\:}_{p}^{{\prime\:}{\prime\:}}+{\epsilon\:}_{c}^{{\prime\:}{\prime\:}}=\frac{{\epsilon\:}_{s}-{\epsilon\:}_{\infty\:}}{1+{\omega\:}^{2}{r}^{2}}\omega\:r+\frac{\sigma\:}{\omega\:{\epsilon\:}_{0}}$$

where $\:{\epsilon\:}_{s}$and $\:{\epsilon\:}_{\infty\:}$ are the permittivitys at static magnetic field and infinite frequency, respectively, $\:{\epsilon\:}_{p}^{{\prime\:}{\prime\:}}$and $\:{\epsilon\:}_{c}^{{\prime\:}{\prime\:}}$ represent the polarization loss and conductance loss of dielectric loss, respectively, $\:\omega\:$ ($\:\omega\:=2\pi\:f$) is the angular frequency, $\:r$ is the relaxation time, $\:\sigma\:$ is the conductivity. For the polarization loss part, the relationship between $\:{\epsilon\:}^{{\prime\:}{\prime\:}}$ and $\:{\epsilon\:}^{{\prime\:}}$ can be described as [52]:

7 $$\:{({\epsilon\:}^{{\prime\:}}-\frac{{\epsilon\:}_{s}+{\epsilon\:}_{\infty\:}}{2})}^{2}+{\left({\epsilon\:}^{{\prime\:}{\prime\:}}\right)}^{2}={\left(\frac{{\epsilon\:}_{s}-{\epsilon\:}_{\infty\:}}{2}\right)}^{2}$$

When there is polarization relaxation in the material, the relationship curve between the $\:{\epsilon\:}^{{\prime\:}}$ and $\:{\epsilon\:}^{{\prime\:}{\prime\:}}$ of the material will appear as a semicircle, that is, a cole-cole semicircle, and every semicircle represents an interface polarization or polarization relaxation process in the material. In order to explore the interface polarization phenomenon in Co_x_Ni_y_@C, the curves of ε ‘and ε ‘’are drawn, as shown in Figs. 6 and 7. It can be seen that there are a large number of semicircles in the cole-cole curves of each sample, which means that there are a large number of interfacial polarization and polarization relaxation processes in each sample, and these polarization may come from the heterogeneous interface between cobalt-nickel and graphite-carbon [53, 54]. Moreover, there is a straight line at the end of each curve, which means that conductance loss is the main loss mechanism at low frequencies. These polarization relaxation processes are important loss modes in dielectric loss of materials, which will make great contribution to the electromagnetic absorption properties of materials.

In the theory of electromagnetic wave absorption, there is a very important influencing factor known as the quarter-wavelength mechanism. During the transmission of electromagnetic waves, when the phase difference between the reflected electromagnetic waves at the air interface and the substrate interface reaches 180°, they will cancel each other out due to the principle of interference cancellation, thereby achieving the effect of attenuating the electromagnetic waves. The relevant formula is as follows [55–57]:

8 $$\:{t}_{m}=nc/4{f}_{m}{\left({\epsilon\:}_{r}{\mu\:}_{r}\right)}^{1/2}$$

In the formula, t_m_ represents the matching thickness of the MAMs, n is a positive odd number, and f_m_ represents the peak frequency. Obviously, one-dimensional samples can achieve specific matching frequencies at lower thicknesses thanks to the enhanced permittivity, which further implies a reduction in the required mass of powder during the coating process. (Figure S1)

The three-dimensional schematic diagram of Co_x_Ni_y_@C absorbing performance is shown in Figs. 6 and 7, among the four samples, Co_3_Ni_1_@C and Co_2_Ni_1_@C have better microwave absorption properties when the thickness is thin. The RL_min_ of Co_3_Ni_1_@C was − 43.2 dB, the EABD was 5.8 GHz (2.1 mm), almost covering the Ku band. At 9.5 GHz, the RL_min_ of Co_3_Ni_1_@C can reached − 45.1 dB (2.6 mm). The strongest absorption of Co_2_Ni_1_@C was − 48.4 dB and the EABD was 4.8 GHz (2.2 mm). When the thickness was further reduced, the EABD was 5.2 GHz (2.1 mm). Figure 6g and h show the microwave absorption performance of Co_3_Ni_1_@C and Co_2_Ni_1_@C at different frequencies and thicknesses in a more intuitive two-dimensional schematic diagram.

Compared with Co_3_Ni_1_@C and Co_2_Ni_1_@C, the performance of Co_3_Ni_2_@C is decreased, the RL_min_ was − 43.1 dB (5.4 mm) at 17.2 GHz. On the one hand, with the increase of Ni content, it can be seen from SEM photos that the aspect ratio gradually decreases, and the effect of forming conductive network is not ideal; On the other hand, according to Fig. 5a and d, it can be seen that Co_3_Ni_2_@C has low permittivity and high permeability, which makes the impedance matching of Co_3_Ni_2_@C poor at low frequency. In Fig. 7c, we can find that the impedance value of Co_3_Ni_2_@C is around 1 when the thickness is about 5.5 mm at high frequency, and the impedance values of other parts deviate from 1. According to Eq. (3) (4), the impedance mismatch between other thicknesses and frequencies leads to the decrease of wave absorption ability of Co_3_Ni_2_@C. The RL_min_ of Co_1_Ni_1_@C is −20.3 dB at 3.1 mm thickness and 8.8 GHz frequency. The EABD is 5.2 GHz at 2.2 mm. At low filling ratio, Co_1_Ni_1_@C has poor microwave absorption performance due to its low permittivity. When the filling ratio of Co_1_Ni_1_@C reaches 30%, its RL_min_ can reach − 42.4 dB.

In order to simulate the electromagnetic absorption ability of Co_x_Ni_y_@C in practical application, the radar cross section (RCS) of the absorber is simulated by CST simulation software in this work, and can be calculated according to the following equation [58]:

9 $$\:\sigma\:\left(dB{m}^{2}\right)=10log{\left(\frac{4\pi\:S}{{\lambda\:}^{2}}\left|\frac{{E}_{s}}{{E}_{i}}\right|\right)}^{2}$$

$\:{E}_{s}$ is the electric field strength of the scattering wave, $\:{E}_{i}$ is the electric field strength of the incident wave, S is the area of the model, and $\:\lambda\:$ is the wavelength.

**Fig. 8:**
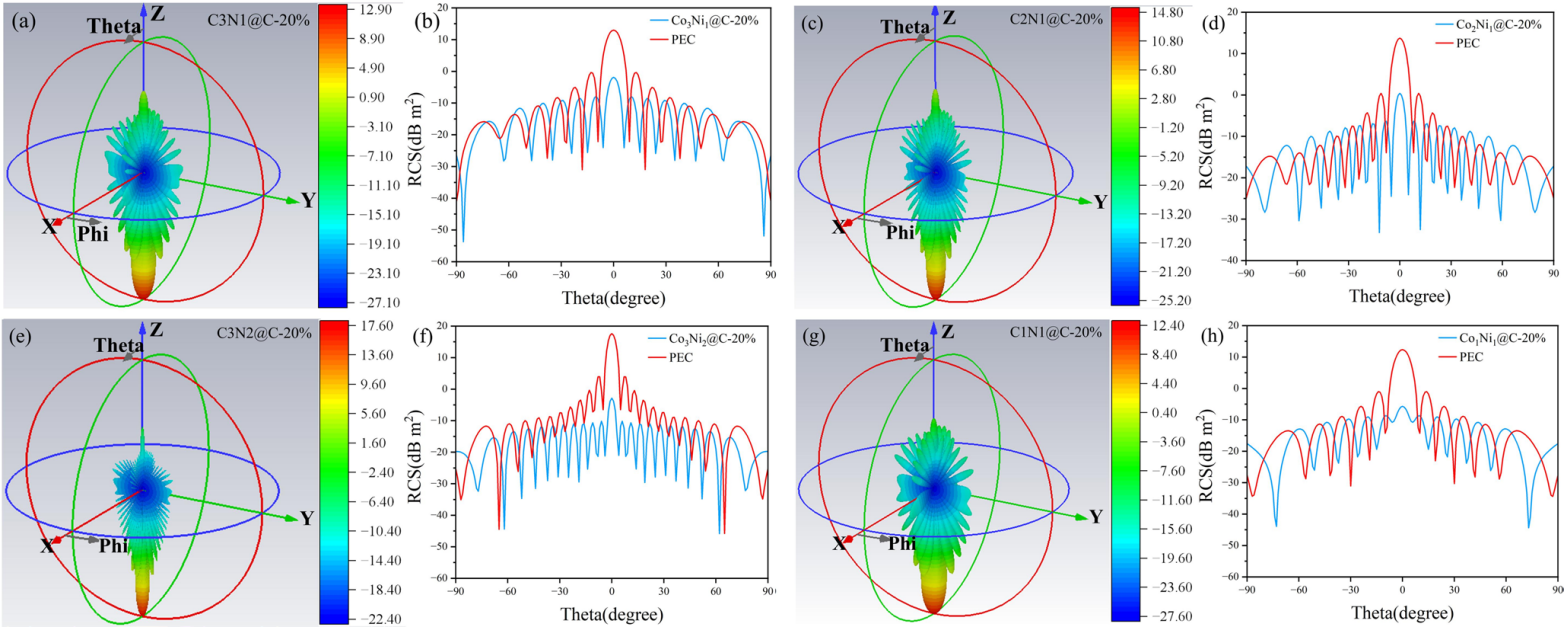
Simulation results of PEC covered with of pea pod-like Co_x_Ni_y_@C with different atomic ratios of cobalt and nickel: (**a**) Co_3_Ni_1_@C, (**c**) Co_2_Ni_1_@C, (**e**) Co_3_Ni_2_@C, (**g**) Co_1_Ni_1_@C. RCS values of each sample in effective angle range (− 90° to 90°): (**b**) Co_3_Ni_1_@C, (**d**) Co_2_Ni_1_@C, (**f**) Co_3_Ni_2_@C, (**h**) Co_1_Ni_1_@C

We built a 180 mm*180 mm square absorbing coating in the X-O-Y plane, the boundary condition of the coating is good conductor, the frequency of the incident wave and the matching thickness of the coating adopt the frequency and thickness corresponding to RL_min_. As shown in Fig. 8, It can be seen from the difference of three-dimensional RCS between upper and lower coatings that one-dimensional Co_x_Ni_y_@C MAMs with different Co and Ni ratios have good electromagnetic absorption ability. On the other hand, in the Y-O-Z plane (Phi = 0), the two-dimensional RCS distribution curve shows radar wave absorption capacity of Co_x_Ni_y_@C and PEC at different electromagnetic wave incident angles. Among them, when the incident angle of Co_3_Ni_1_@C is 86°, the minimum RCS value can reach − 53.74 dB·m^2^, as shown in Fig. 8b. Co_2_Ni_1_@C has a minimum RCS of −33.26 dB·m^2^ at an incident angle of 12°, as shown in Fig. 8d. The minimum RCS of Co_3_Ni_2_@C can reach − 44.39 dB·m^2^ at an incident angle of 62°, as shown in Fig. 8f. Co_1_Ni_1_@C has a minimum RCS of −43.96 dB·m^2^ at an incident angle of 73°, as shown in Fig. 8h. The RCS of Co_x_Ni_y_@C is less than 0 in all incident angles from − 90° to 90°. It can be seen that Co_x_Ni_y_@C has certain practical application value.

## Antioxidant test

In order to verify whether the oxidation resistance of the materials was improved by adding Ni, Co_x_Ni_y_@C MAMs with different ratio of Co and Ni were oxidized at 350 ℃ for 12 h in air atmosphere. The oxidized samples were analyzed by XRD and their absorbing properties were tested.

**Fig. 9:**
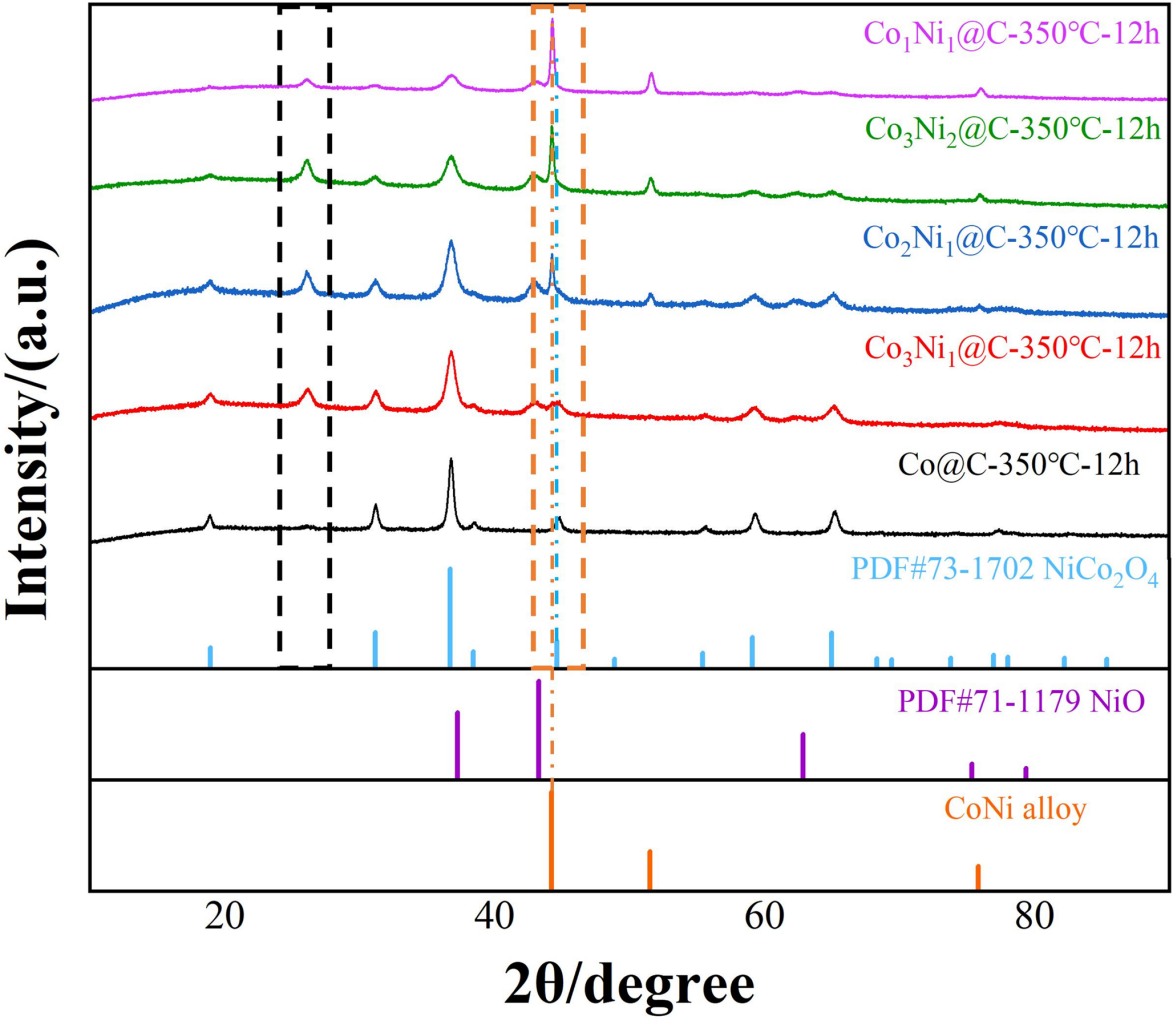
XRD patterns of pea pod-like Co_x_Ni_y_@C MAMs with different Co and Ni ratios after oxidation treatment at 350℃

The XRD spectra obtained are shown in Fig. 9. It can be seen that after oxidation at 350 ℃ for 12 h, the internal metal particles of Co@C nanotubes have been completely oxidized into spinel Co_3_O_4_ phase, and other Co_x_Ni_y_@C nanotubes with different Ni contents also have different degrees of oxidation, but they all maintain a certain amount of alloy phase, and with the increase of Ni content, the alloy phase content maintained in Co_x_Ni_y_@C also increases, which proves that adding Ni to internal magnetic alloy particles can improve the oxidation resistance of MAMs. The black dotted box in Fig. 9 corresponds to the characteristic peak of graphite, and its relative strength is higher than that before oxidation treatment, which may be due to the further increase of graphitization degree of graphite layer under the action of high temperature.

**Fig. 10:**
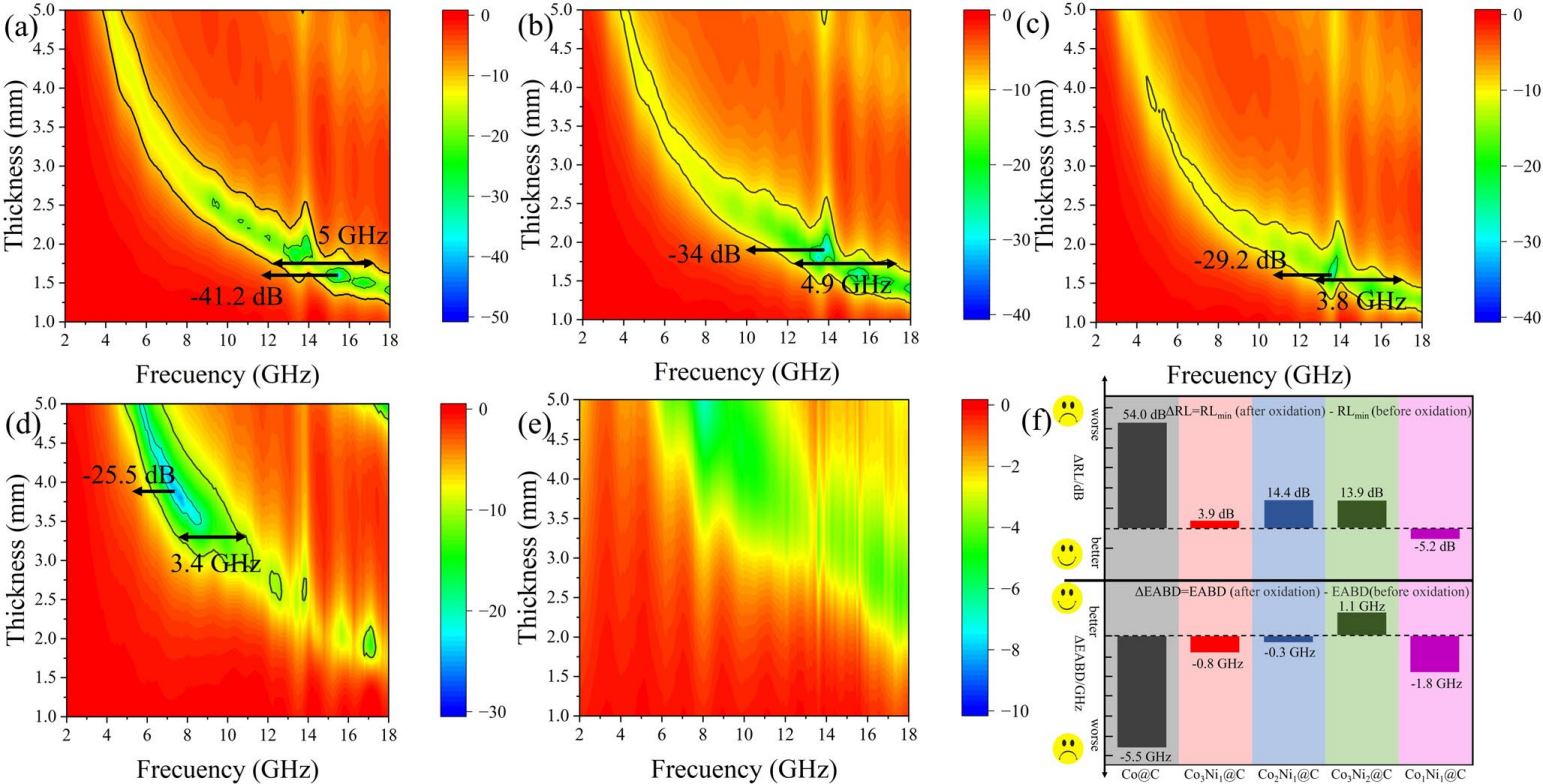
3D RL maps of pea pod-like Co_x_Ni_y_@C MAMs with different Co and Ni ratios after oxidation treatment at 350℃: (**a**) Co_3_Ni_1_@C, (**b**) Co_2_Ni_1_@C, (**c**) Co_3_Ni_2_@C, (**d**) Co_1_Ni_1_@C, (**e**) Co@C. (**f**) Comparison chart of changes in absorbing properties of Co@C and Co_x_Ni_y_@C before and after oxidation

The microwave absorbing properties of Co_x_Ni_y_@C nanotubes after oxidation are shown in Fig. 10. After oxidation at 350 ℃ for 12 h, the RL_min_ and EABD of Co_3_Ni_1_@C at 15.5 GHz and 1.6 mm were − 41.2 dB and 5 GHz respectively; Co_2_Ni_1_@C at 13.8 GHz, 1.9 mm RL_min_ is −34 dB, EABD 4.9 GHz; Co_3_Ni_2_@C at 13.6 GHz, 1.6 mm RL_min_ is −29.15 dB, EABD is 3.8 GHz; The RL_min_ and EABD of Co_1_Ni_1_ @ C at 7.7 GHz and 3.8 mm were − 25.5 dB and 3.4 GHz respectively. After oxidation at 350 ℃, the RL_min_ of Co_3_Ni_1_@C and Co_1_Ni_1_@C changed little, and the RL_min_ of Co_1_Ni_1_@C increased slightly, but the RL_min_ of Co_2_Ni_1_@C and Co_3_Ni_2_@ C decreased, and all the samples with four ratios of EABD decreased, but in the range of 2–18 GHz, the EABD of Co_3_Ni_2_@C is 1.1 GHz wider than before oxidation. In order to reflect the effect of Ni addition on the oxidation resistance of the material, we prepared Co@C one-dimensional MAMs in previous work [59], the RL_min_ is-61 dB and the EABD is 5.5 GHz (1.94 mm), which has excellent absorbing performance, but after undergoing oxidation treatmen, the absorbing performance almost disappears. The changes of RL_min_ and EABD before and after oxidation of Co@C and Co_x_Ni_y_@C are shown in Fig. 10f. According to the degree of performance change, it can be clearly seen that Co_x_Ni_y_@C has good oxidation resistance. Generally speaking, after oxidation treatment, one-dimensional Co_x_Ni_y_@C nanotubes still have good electromagnetic absorption properties. Due to the addition of Ni, an oxide layer is formed on the surface of the alloy particles, which protects the internal particles from further oxidation. It provides a new idea for the follow-up study on the oxidation resistance of MAMs at high temperature.

Table S1 summarizes the comparison of the electromagnetic wave absorption properties of the materials we prepared with those of other carbon-based magnetic composite MAMs that have been reported [60, 61]. It can be observed that, although the composite materials we prepared perform averagely in terms of reflection loss, they exhibit comprehensive excellent performance in EABD, matching thickness and filler content. Combined with the excellent performance of the antioxidant test, we believe that the prepared composite MAMs demonstrate reliable practical application value and can provide guidance for the future development of MAMs.

**Fig. 11:**
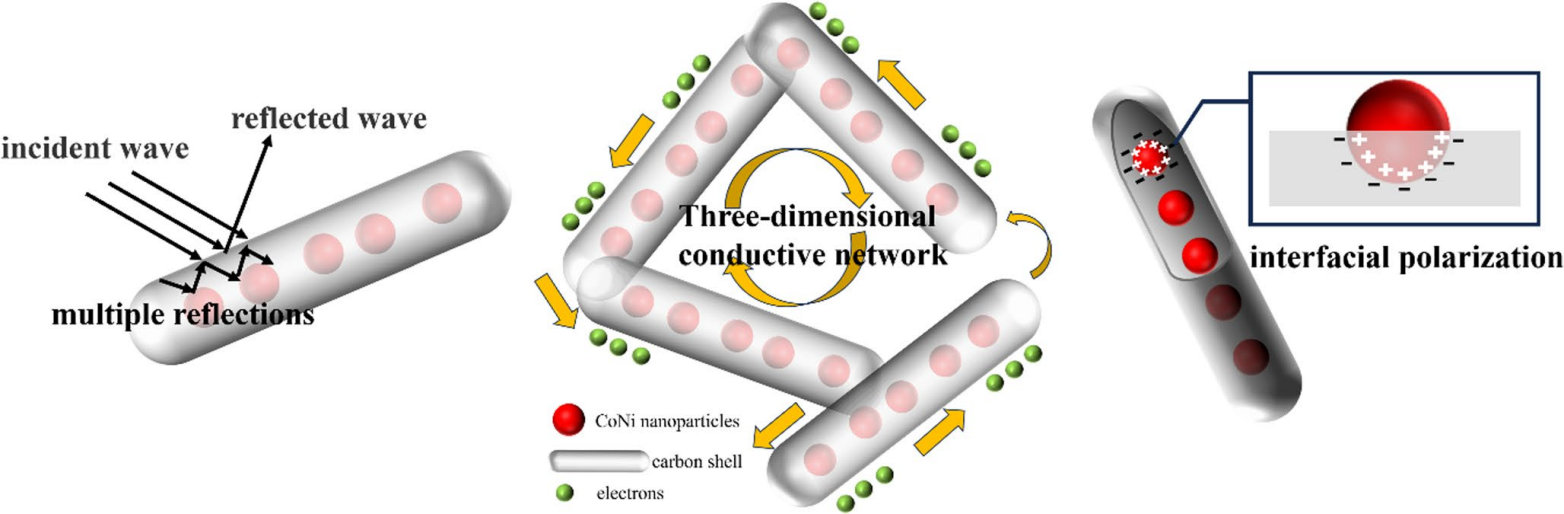
Attenuation mechanisms of microwave absorption of pea pod-like Co_x_Ni_y_@C

To sum up, the absorbing mechanism of one-dimensional core-shell Co_x_Ni_y_@C electromagnetic absorbing material is shown in Fig. 11, which can be summarized as follows: (1) Co_x_Ni_y_@C composites magnetic nanoalloy particles with carbon materials, in which the carbon shell provides the main dielectric loss, and the internal magnetic alloy particles balance the impedance while providing part of the magnetic loss. (2) One-dimensional particle structures overlap each other to form a three-dimensional conductive network, which is helpful to the migration and jumping of electrons, enhance the conductivity loss of materials, and achieve good impedance matching at a lower filling rate, so that materials have the advantages of lightweight on the basis of good wave absorbing ability. Based on the Debye free electron theory, the conductive loss mainly contributes to the imaginary part of the dielectric constant curve at low frequencies. Combining theoretical analysis with characterization data, the conductive loss contributed by the one-dimensional nanostructure of the composites may dominate in the dielectric loss [62, 63]. (3) Because of the core-shell structure, there are a large number of heterogeneous interfaces in the material, and a large number of positive and negative charges will gather on both sides of the heterogeneous interfaces, resulting in polarization relaxation of the interface polarization under the action of electromagnetic waves, which enhances the energy loss of the material to electromagnetic waves [64–66]. (4) Due to the characteristics of the preparation process, there are a large number of point defects in the composite materials, such as N, O impurity atoms and vacancies in the carbon layer. The existence of these defect forms alters the distribution of electron cloud, disrupts the local charge balance, and leads to the emergence of electric dipoles. Under the action of an externally applied high-frequency electromagnetic field, the electric dipoles exhibit the Debye relaxation phenomenon, converting electromagnetic energy into heat and dissipating it. Interface polarization and dipole polarization mainly contribute to the dielectric loss capability in the mid-high frequency range to balance the impedance matching in this frequency range [67–69]. (5) The core-shell structure provides multi-layer interface and cavity, which is helpful for electromagnetic waves to have multiple reflections in the interior to lose energy.

## Conclusion

Overall, we prepared pea pod-like Co_x_Ni_y_@C one-dimensional core-shell MAMs with self-adjusting atomic ratio, which provided a simple and convenient preparation method for preparing MAMs with personalized atomic ratio. The conductive network formed by core-shell structure and one-dimensional structure can enhance the interface polarization and conductivity, so that the material can achieve good electromagnetic absorption performance at a low filling rate of 20%. The RL_min_ of Co_x_Ni_y_@C can reach-48. 4 dB, and the EABD can reach 5.8 GHz. In addition, Co_x_Ni_y_@C obtained different degree of oxidation resistance by adding different proportion of Ni. After oxidation treatment at 350 ℃ for 12 h, Co_3_Ni_1_@C could still maintain an absorption strength of −41.2 dB and an effective absorption width of 5 GHz. We believe that the content of this work has certain application value in special fields, and has certain guiding significance for the follow-up research on lightweight and antioxidant capacity of MAMs.

## Supplementary Information

Below is the link to the electronic supplementary material.

Supplementary Material 1 (DOCX 124 KB)

## Data Availability

No datasets were generated or analysed during the current study.
