# Supplementary material for: One-dimensional well-defined peapod CoxNiy@C nanocomposite hollow nanorod towards enhanced electromagnetic performance
Source: Adv Compos Hybrid Mater. 2026 Mar 2;9(2):146. doi: 10.1007/s42114-026-01702-0 (PMC12999744; doi:10.1007/s42114-026-01702-0)
Supplement: Supplementary file 1 — Supplementary Material 1 (DOCX 124 KB) [file 42114_2026_1702_MOESM1_ESM.docx]

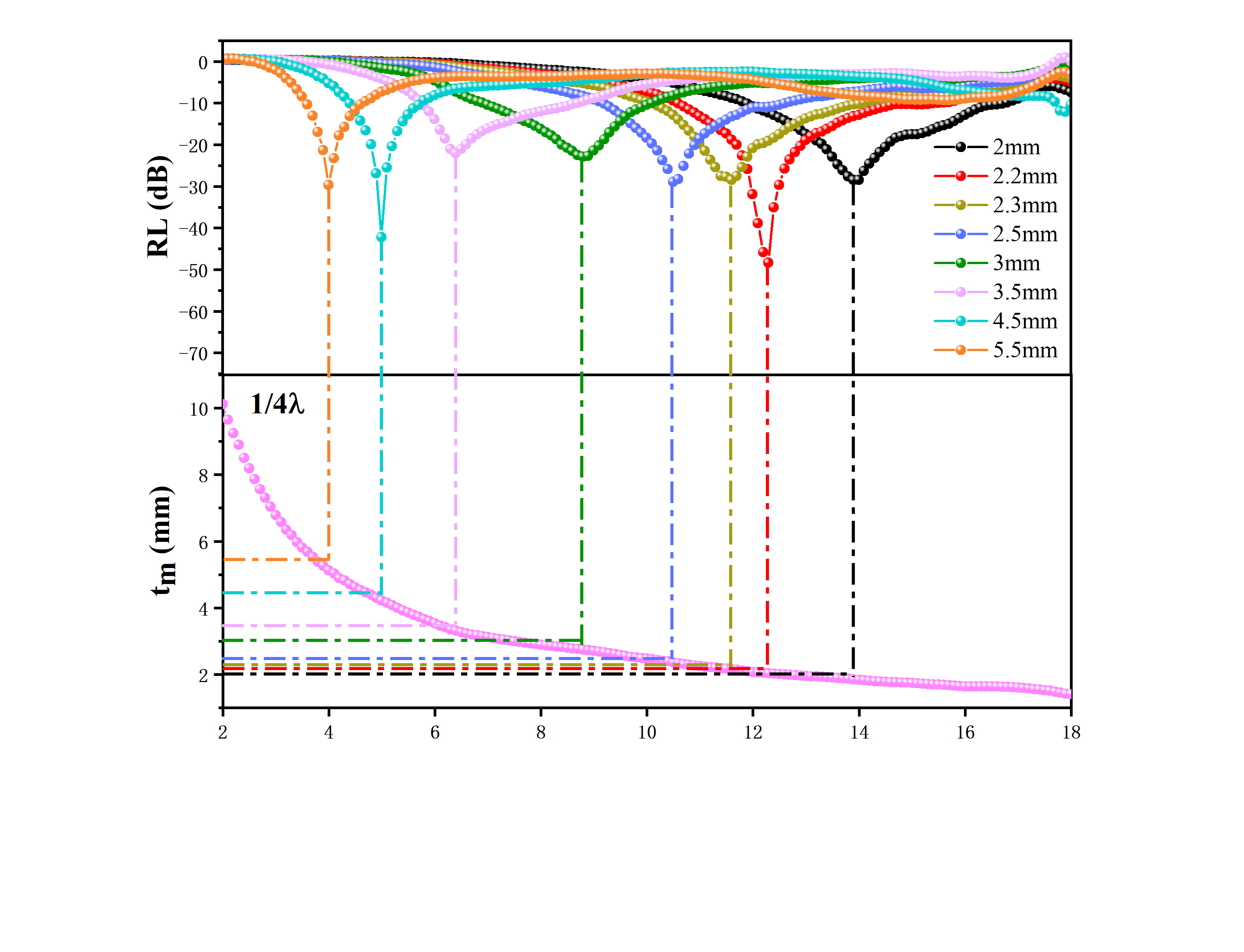


Figure S1 RL curves and matching thickness curves of Co_2_Ni_1_@C

Table S1. A comparison table of microwave absorption performance of some carbon-based magnetic composite in the previous reports.

| Sample | RL_min_ (dB) | EABD (GHz) | Filler content (%) | d_m_ (mm) | Ref. |
| --- | --- | --- | --- | --- | --- |
| CMS/Ni/PVDF | -90.29 | x-band | 50 | 1.5 | 60 |
| PCPS/Co-Zn | -96.55 | 4.0 | 10 | 2.25 | 61 |
| Co_2_Ni_1_@C | -48.4 | 4.8 | 20 | 2.2 | This work |
| Co3Ni1@C | -43.2 | 5.8 | 20 | 2.1 | This work |
